# Evaluation of a multi-component prevention concept for hazardous substance use among refugees in shared accommodations: study protocol for a cluster randomized controlled trial

**DOI:** 10.1186/s13063-024-08558-z

**Published:** 2024-11-15

**Authors:** Miriam Hedwig Lorenz, Jonathan Uricher, Markus Iwan Pauzar, Johannes Michalak, Marion Laging, Thomas Heidenreich

**Affiliations:** 1 Faculty of Social Work, Education and Nursing Sciences, Esslingen University of Applied Sciences, Flandernstraße 101, Esslingen, 73732 Germany; 2 Dept. of Psychology and Psychotherapy, Witten/Herdecke University, Alfred-Herrhausen-Straße 50, Witten, 58448 Germany

**Keywords:** Evaluation, Cluster randomized controlled trial, Stepped-wedge-design, Hazardous substance use, Prevention, Refugees, Forced migration

## Abstract

**Background:**

Refugees are exposed to various risk factors in shared accommodations in Germany where they are housed after their arrival. Due to their often traumatic experiences before, during, and after their flight and socio-structural post-migration stressors, refugees are potentially vulnerable to hazardous substance use. They form a structurally disadvantaged group for substance use prevention and intervention. Various barriers make it difficult for them to access the healthcare system and to get health information. Therefore, a participatory multi-component prevention concept for refugees in shared accommodations was developed. The aim of the trial is to evaluate the efficacy of this concept regarding the increase in knowledge about substance use among refugees living in shared accommodations.

**Methods:**

Based on a randomized controlled stepped-wedge design, the study will be conducted in a multicenter setting in nine shared accommodations and will aim to include refugees living in shared accommodations as well as social workers and psychologists working there.

**Discussion:**

This trial will be one of the first to generate evidence about effective participatory prevention concepts for refugees in shared accommodations. Evidence-based concepts for refugees can improve access to health care and might be helpful for the multidisciplinary workforce in shared accommodations.

**Trial registration:**

OSF Registry: osf.io/ebnj3. Registered on May 24, 2024. Registration DOI: https://doi.org/10.17605/OSF.IO/EBNJ3.

## Administrative information

**Table:** We used the structured study protocol template (https://trialsjournal.biomedcentral.com/submission-guidelines/preparing-your-manuscript/study-protocol). Numbers refer to the according sections in Trials-/SPIRIT-checklist

Title {1}	Evaluation of a Multi-Component Prevention Concept for Hazardous Substance Use Among Refugees in Shared Accommodations: Study Protocol for a Cluster Randomized Controlled Trial
Trial registration {2a and 2b}.	OSF Registry: osf.io/ebnj3. Registered May, 24, 2024, Registration DOI: https://doi.org/10.17605/OSF.IO/EBNJ3
Protocol version {3}	08/2024; version 2
Funding {4}	The German Federal Ministry of Education and Research supported this work (research and development project PraeWi; funding reference: 13FH045SX7).
Author details {5a}	**¹ **Hochschule Esslingen University of Applied Sciences Faculty of Social Work, Education and Nursing Sciences Flandernstraße 101 73732 Esslingen Germany miriam.lorenz@hs-esslingen.de jonathan.uricher@hs-esslingen.de m.pauzar@stud.hs-esslingen.de marion.laging@hs-esslingen.de thomas.heidenreich@hs-esslingen.de **² **Witten/Herdecke University Dept. of Psychology and Psychotherapy Alfred-Herrhausen-Straße 50 58448 Witten Germany johannes.michalak@uni-wh.de
Name and contact information for the trial sponsor {5b}	German Federal Ministry of Education and Research; research and development project PraeWi (Funding reference: 13FH045SX7; "FH-Sozial 2017: Präventionsmaßnahmen und Wissenstransfer innerhalb der Sozialen Arbeit bezüglich riskanten Substanzkonsums für Menschen mit Fluchterfahrungen in Übergangswohnheimen (PraeWi)"). Contact information: Bundesministerium für Bildung und Forschung 53170 Bonn; 11055 Berlin;www.bmbf.de;+49 (0)228 99 57-2728
Role of sponsor {5c}	The research and development project PraeWi is sponsored by German Federal Ministry of Education and Research and located at University of Applied Sciences Esslingen. Responsibility for execution of the project is transferred to the local project management. The project management is responsible for the design of the study, the data collection, management, analysis and interpretation of the data, and the writing and submitting of the report about the study.

## Introduction

### Background and rationale {6a}

The UNHCRs Mid-year Trends Report [38] states that 110 million people are forcibly displaced within or outside their country of origin (UNHCR, [38]). As one of the three major hosting countries in the world, Germany hosted approximately 2.5 million people seeking international protection in mid-[12], who had to leave their home due to persecution, war, the consequences of climate change, dictatorships, poverty, and famine, with numbers increasing and expected to further increase over the years (UNHCR, [38]). Owing to their often traumatic experiences before, during, or after their flight, refugees are vulnerable to hazardous substance use and might use substances to cope with their psychological distress. The World Drug Report [40] highlights refugees as a vulnerable group for hazardous substance use due to their experiences of social and mental health issues in humanitarian settings and in their post-displacement environment (UNODC, [39]a; [40]b). In Germany, refugees are temporarily housed in shared accommodations. In these accommodation facilities, refugees are exposed to various socio-structural risk factors and post-migratory stressors [20, 41]. In addition, refugees face various barriers to existing substance use interventions and to adequate information about induced health risks [23, 28, 31, 42, 44]. Therefore, there is a need for culturally sensitive and low-threshold prevention measures that give health-related information, reduce the need for intervention, and can be applied at an earlier stage during individuals’ stay in shared accommodations.

While much is known about forced migration on the one hand and substance abuse on the other hand, little is known about hazardous substance use among refugees. There is some evidence about the epidemiology of substance use among refugee populations [15, 17, 44] and about substance use prevention among refugees (summarized by [19], however, there is no evidence about effective evidence-based concepts to prevent substance use among refugees in the setting of shared accommodations. The existing findings from other target groups are only partially transferable to this specific group of refugees and their living environment [18], which is characterized by sharing rooms, lack of privacy, limited space, and the cohabitation of many different people from different countries, cultures, and religions.

Specific, culturally sensitive, adequate, and evidence-based prevention concepts containing access to information are urgently needed but have not yet been developed and evaluated. Therefore, the aim of this trial is to evaluate the effects of a multi-component prevention concept for refugees in shared accommodations in Germany, which has been developed in a participatory way with refugees, social workers, and researchers.

### Objectives {7}

The primary objective of this study is to evaluate the efficacy of a multi-component prevention concept for refugees in shared accommodations regarding refugees’ knowledge about hazardous substance use and induced health risks. It is hypothesized that their knowledge about the health system, addiction counselling, risks of hazardous substance use, and access to supportive structures increases in shared accommodations where the concept is implemented (intervention group) in comparison to the shared accommodations where the concept is not yet implemented (control group). The secondary objectives are to measure the impact of concept implementation on health literacy, attitudes towards substance use, hazardous substance use, depression, anxiety, and stress as well as the knowledge of the workforce about hazardous substance use and induced health risks.

### Trial design {8}

The study is based on a randomized controlled stepped-wedge design and will be conducted in a multicenter setting in nine shared accommodations. The stepped-wedge design ensures that the prevention concept is available to all refugees in the nine shared accommodations in the final phase of the study. In addition, the design enables a deeper analysis of the effect over time, which a conventional cluster randomization would not offer. Randomization is planned for the allocation of the shared accommodation to the intended clusters A, B, and C in three steps (step 1: intervention in cluster A; step 2: intervention in clusters A and B; and step 3: intervention in clusters A, B, and C).

## Methods: participants, interventions, and outcomes

### Study setting {9}

The study will take place in nine shared accommodations in Stuttgart, Germany. The survey will consist of three clusters with three accommodations each. Using the web-based randomizing program random.org, shared accommodations are randomly assigned to each cluster. Implementation takes place in cluster A in weeks 1–3, cluster B in weeks 4–6, and cluster C in weeks 7–9. This results in five survey times t_0_–t_4_ (see Table 1): baseline, after implementation in clusters A, B, and C as well as the follow-up. In the study, data is collected and evaluated both at the level of the refugees and at the level of the qualification of the setting (qualification of the professionals in the shared accommodation, structural conditions of the setting). Qualified social workers or psychologists perform the implementation of the prevention concept.

**Table 1:**
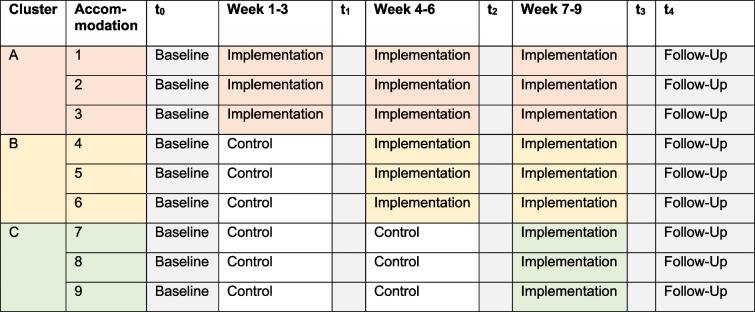
Trial design

### Eligibility criteria {10}

Inclusion criteria for the participants are (1) being a refugee or asylum seeker or workforce in shared accommodations; (2) residing or working in selected shared accommodations; (3) being 18 + years, and (4) language proficiency (oral/written) in the languages in which the survey instrument is available (Arabic, Turkish, Farsi, Russian, French, German, and English). Exclusion criteria are (1) persons in acute mental health crises (e.g., suicidality, psychosis) and (2) minors.

The inclusion criteria (1), (2), (3), and (4) are tested by members of the organizational support groups before inviting participants to the trial. Members of the organizational support groups know the residents of shared accommodations very well by providing them day-to-day support and social services. They also have access to all relevant medical and organizational files. Assessment of exclusion criterion (1) (acute mental health crisis) is also performed by members of the organizational support groups by behavioral observation in the shared accommodations on a daily basis. Values of 30 or lower on the Global Assessment of Functioning Scale [1], of the DSM-IV are considered to be indicators of acute mental health crises. To our knowledge, no formal measures for objectivity, reliability, and validity of the GAF for residents of shared accommodations for refugees are available; however, it is assumed that day-to-day interaction of staff and refugees is perfectly suited to detect serious impairment as described above. Apart from the nine included shared accommodations, there are specific shared accommodation facilities in which only refugees with diagnosed acute mental crises and mental illness are housed. These so-called protective facilities are deliberately excluded from the sampling to ensure the safety of the participants. As a result, it is initially unlikely that a person with a diagnosed mental illness will be invited to participate in the study. Exclusion criterion (2) (age below 18) is also rated by members of the organizational support groups based on organizational files.

### Who will take informed consent? {26a}

The research team on site and the organizational support groups provide information and flyers about the purpose of the study and how to participate in different languages (Arabic, Turkish, Farsi, Ukrainian, Russian, French, German, and English). If they meet the eligibility criteria and are willing to participate, participants can give written consent and agree to the use of their data. Formal informed consent is given before starting to fill in the questionnaire online.

### Additional consent provisions for collection and use of participant data and biological specimens {26b}

This is not applicable because no biological specimens will be collected in this trial.

## Interventions

### Explanation for the choice of comparators {6b}

The comparators are shared accommodations, in which the intervention was not yet implemented according to the stepped-wedge design. Shared accommodations that are waiting for the intervention will not be offered any additional interventions.

### Intervention description {11a}

To address refugees as a particularly vulnerable group for hazardous substance use, the research and development project “PraeWi” (funded by the German Federal Ministry of Education and Research) developed a participatory multi-component prevention concept containing different addiction prevention measures for the workforce and for refugees working or living in shared accommodations (see Table 2).

**Table 2:** Multi-component prevention concept

Components	Prevention measures
1. Health communication through peers	Information videos
	Peer education
2. Tools for the workforce	Guide with situational recommendations
	E-learning-tool for the workforce
3. Bridges in support system	Linking the specialist areas Flight—Trauma—Addiction
4. Online prevention	Podcast
	BePrepared-App
5. Target group-specific information and communication strategy	Communicating the project results to different population groups/stakeholders in a tailored target group approach

This concept provides cultural sensitive information videos in different languages (Arabic, Turkish, Farsi, Ukrainian, Russian, French, German, and English), a podcast, an app and material for peer education workshops for refugees, and an e-learning tool and guidelines for the workforce of shared accommodations. The prevention measures of components 1–4 will be provided via QR codes that give access to the project website where all the material can be downloaded. The psycho- and socio-educative materials include information about the health system, addiction counselling, risks of hazardous substance use, and access to supportive structures. Component 5 is a superordinate component and therefore not part of the intervention.

The concept is based on an acceptance-oriented perspective to promote risk awareness and competence as well as health literacy [2, 11, 14, 43]. It was developed in a participatory way. Moreover, the study will be conducted with refugees participating in the research and implementation team.

Since the intervention is targeting several shared accommodations (each with a number of refugees), adherence to protocol is not assessed on an individual level. However, on the level of each shared accommodation, adherence will be checked by the project management group by making sure that all prevention materials are available in the study centers. As the intervention material consists of 4 prevention components that are made available at each study center (component 5 is of a more global nature), adherence scores rated by members of the project management group can range from 0 (no materials available) to 4 (all 4 materials available).

### Criteria for discontinuing or modifying allocated interventions {11b}

Participants are free to use or not use the prevention measures. In case of serious adverse events, the installed psychological background service will be consulted and the project steering committee will decide whether the study should be discontinued. As the focus of the concept is prevention, no worsening of diseases will be expected.

### Strategies to improve adherence to interventions {11c}

The organizational support groups, composed of social workers and psychologists in shared accommodations, will encourage participants to use prevention materials. Due to their participatory development, these materials are more sensitive to language and culture. The organizational support groups are qualified for target group-specific, culturally sensitive language and are competent to use specific communication strategies to encourage participants.

### Relevant concomitant care permitted or prohibited during the trial {11d}

Concomitant care is permitted but is not foreseen during the trial.

### Provisions for post-trial care {30}

As the intervention implemented during this trial is a prevention concept and participants are free to participate, we do not expect that harm will occur to trial participants. Nonetheless, they can contact the research team until the project is finished at the end of March 2025.

### Outcomes {12}

The primary outcome is the increase of knowledge about substances, hazardous substance use, and induced health risks among refugees since baseline. This will be measured with the Drug and Drug Problems Perception Questionnaire (DDPPQ; [4] in an adapted version to test the efficacy of the prevention measures in providing health-related information creating a sum score for a continuous variable. Primary outcome will be measured at the study times t_0_–t_3_.

The following short-term secondary outcomes are assessed 9 weeks after the first implementation: health literacy measured with eHealth literacy scale (eHeals; (Norman et al. [24, 25]), attitudes towards substance use measured with an adapted version of the Substance Abuse Attitude Survey (SAAS; [8]), and the increase in knowledge of the workforce about substances, hazardous substance use, and health risks among refugees measured with an adapted version of the Drug and Drug Problems Perception Questionnaire (DDPPQ; [4]) related to the intervention.

In order to relate the outcomes directly to the intervention, the DDPPQ; [4] and the SAAS [8] were adapted to the intervention and the context of the concept implementation. As in other studies [9, 22], where the DDPPQ was also adapted to specific contexts, statements about the validity of the scales need to be tested in a context-specific way.

The following long-term secondary outcomes are assessed three months after first implementation: depression, anxiety, and stress measured with Depression Anxiety Stress Scale (DASS–21; [21]) and reduction of hazardous substance use measured with DSM-5 criteria of substance use disorder [5], Obsessive Compulsive Drug Use Scale-5 score (OCDUS-5; [13], 30-day prevalence of hazardous substance use, and a self-assessment of problematic substance use [33].

Control variables are socio-demographic data (e.g., age, gender, and nationality) and contextual factors (e.g., accommodation size).

### Participant timeline {13}

Potential participants who reside in the selected shared accommodations will be invited to participate in the study after screening for eligibility. Information about the aims of the study and the possibility of asking questions will be provided by the research team and with multilingual information material. Further on, this material provides information about the funding, data management and protection, anonymization, confidentiality, estimated time to participate, incentives, freedom to withdraw at any time, and contact details. After giving informed consent, potential participants are invited to the baseline data collection (t_0_). Table 3. shows the detailed participant timeline. Baseline assessment will consist of sociodemographics, contextual factors, and primary and secondary outcomes (see outcomes). Afterwards, shared accommodations will be allocated into cluster A, B, or C. In cluster A, the intervention starts 1 week after baseline. According to the stepped-wedge design, implementation steps from one to another cluster take 3 weeks with an invitation to the participants for short assessments regarding primary and short-term secondary outcomes at the end of each implementation step (*t*_1_–*t*_3_). Ten weeks after baseline, the intervention ends in all clusters. Three months after baseline, participants are invited for follow-up to assess long-term secondary outcomes.

**Table 3.**
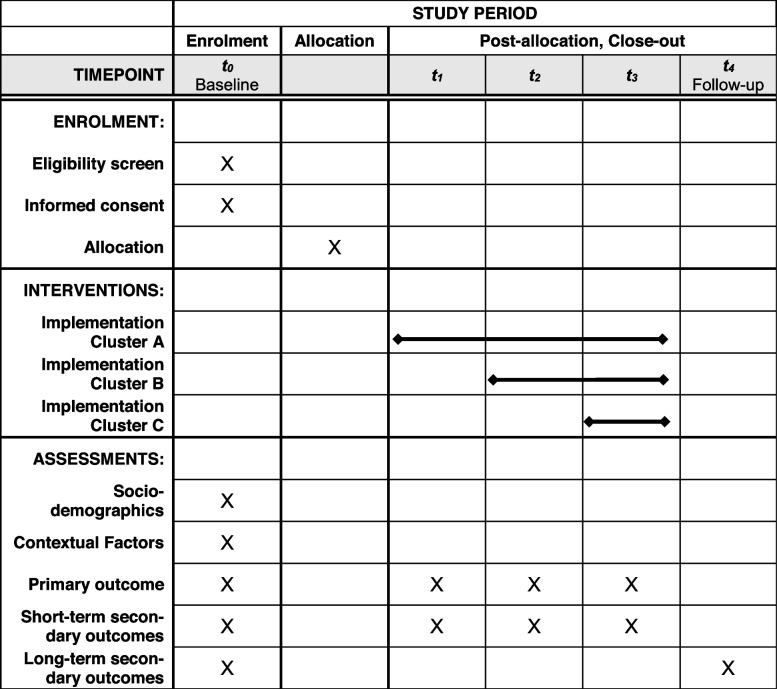
Participant timeline

### Sample size {14}

Following the recommendation of Ouyang et al. [27], the calculation of the estimated number of participants is based on Hemming et al. [16]. Using the described trial design as a stepped-wedge design with a cohort sample structure and an exchangeable correlation structure, we allow varying cluster sizes. According to our experiences with previous research in this field, we assume a coefficient of variation of cluster sizes of 10 participants, mean difference of 0.15, and an intra-class correlation (ICC) of 0.01. Regarding the assumption to reach a statistical power of 0.8 with a significance level of 0.05, the estimated number of participants needed to achieve study objectives is *n* = 252 (28 each shared accommodation in the clusters).

### Recruitment {15}

Shared accommodations are selected where there is a balanced resident structure in terms of country of origin, age, gender distribution, and family situation. Attention is also paid to the inclusion of different sizes and forms of housing (e.g., system construction, existing buildings, etc.). The sample will be drawn using the respondent driven sampling (RDS) method, a further development of snowball sampling, and like this is suitable for populations that are hard to reach [7, 30, 35, 36] for the researchers but whose members are well networked and enables weighting in the analysis through better control of the recruitment channels in order to ensure representativeness of the sample. Participants are invited to take part in the trial by the organizational support groups and with multilingual information material (the following languages are available: Arabic, Turkish, Farsi, Ukrainian, Russian, French, German, and English). During the conduct of the trial, the organizational support groups will make sure that participants continue in the trial by regularly reminding them to participate.

## Assignment of interventions: allocation

### Sequence generation {16a}

The sequence generation for this study's stepped-wedge design will be conducted considering ethics and practical feasibility. The order of interventions will be generated using a web-based random allocation method (random.org) to minimize biases and ensure fair distribution of the intervention across the clusters and its shared accommodations. This sequence generation will be performed by a member of the University Witten/Herdecke who will not be part of the data collection and analysis process to ensure appropriate randomization. Each of the nine shared accommodations will be randomized into one of the three clusters. Afterwards, the three clusters will be randomized to the three sequences according to the stepped-wedge design, which determines the point in time of the intervention.

### Concealment mechanism {16b}

Intervention allocation will be given to the research team after baseline. Therefore, researchers, workforce, and refugees responsible for implementing the interventions will only gain access to the relevant information after the sequence is determined.

### Implementation {16c}

The implementation of the prevention concept will be carried out by a multidisciplinary team of professionals, including researchers of the study team, social workers, and psychologists as well as refugees living in shared accommodations. Staff and peer training will be conducted beforehand to ensure consistent and standardized implementation. The implementation of the prevention concept is developed in close collaboration with residents of shared accommodations to ensure high acceptance and effectiveness.

## Assignment of interventions: blinding

### Who will be blinded {17a}

This is not applicable because blinding is not possible for this specific study.

### Procedure for unblinding if needed {17b}

Not applicable, see 17a.

## Data collection and management

### Plans for assessment and collection of outcomes {18a}

The trained research team is responsible for organizing and conducting the data assessment. Data will be assessed using a multilingual questionnaire containing different item blocks and psychometric scales. The multilingual questionnaire is based on the Measurements in the Addictions for Triage and Evaluation Questionnaire (MATE-Q; [32, 33] and supplemented with other scales and items to measure primary and secondary outcomes. MATE-Q was chosen because of its validity [26], its availability in different languages (e.g., Farsi, Arabic, Turkish, French and Russian), and the possibility of its easy self-completion [33]. It contains questions about substance use and gambling, 30-day prevalence, andlifetime prevalence, the Obsessive Compulsive Drug Use Scale-5 (OCDUS-5; [13]), and items aligned with the DSM-5 substance use disorder criteria [5]. Further on, MATE-Q asks for information about mental and physical well-being, distress, comorbid mental disorders, and ICF-related items addressing problems with relationships and basic requirements. It also contains the Depression Anxiety Stress Scale (DASS–21; [21]).

Further scales not included in the MATE-Q were supplemented. These supplemented scales are a migration-sensitive version of the MacArthur scale [3] based on Schumann et al. [34] to measure subjective social status and the eHealth Literacy Scale (eHeals, [24]) to assess health literacy regarding health information on the Internet. The questionnaire was further enhanced with self-created items to assess sociodemographic and contextual factors as well as with an adapted version of the Drug and Drug Problems Perception Questionnaire (DDPPQ [4, 22]), to measure the knowledge about substance use, an adapted version of the Substance Abuse Attitude Survey (SAAS; [8]), and questions about the satisfaction regarding the living situation based on the German Socio-Economic Panel study (TNS Infratest Sozialforschung, [37]). Participants can gain access to the questionnaire via QR codes for each language (Arabic, Turkish, Farsi, Russian, French, German, and English).

### Plans to promote participant retention and complete follow-up {18b}

Participants will be motivated to participate and to continue participation in the study during the data collection period by incentives (gift cards) and by reminders from the research team and are encouraged by the workforce on site to reduce drop-out and to minimize missing data.

### Data management {19}

Data management will be performed at University of Applied Sciences Esslingen. Data will be collected using EvaSys (data software) and analyzed using R [29].

### Confidentiality {27}

Personal information about the participants will be stored securely at University Esslingen and will be accessible only for the research team. Participants’ data from the questionnaire and the documents that contain the name and signature of the participants will be stored separately. In the run-up to the study, the research team worked closely with the data protection officer and developed a data protection concept. In particular, procedures were developed for the confidentiality of stored data and documents as well as the handling of personal and personally identifiable data. The data protection concept created implements all regulations stipulated by the EU General Data Protection Regulation (EU GDPR) and the Baden-Württemberg State Data Protection Act (LDSG BW, new). Only accumulated and anonymized data will be available for publication after the trial.

### Plans for collection, laboratory evaluation, and storage of biological specimens for genetic or molecular analysis in this trial/future use {33}

This is not applicable because no biological specimens will be collected in this trial.

## Statistical methods

### Statistical methods for primary and secondary outcomes {20a}

For the primary outcome, multilevel linear regression analyses will be employed. The variation of the effect by cluster and over time will be examined [10]. For secondary short- and long-term outcomes, depending on the variable, both multilevel linear and multilevel logistic regression analyses will be used. These methods are suitable for analyzing clustered data, which is inherent in the stepped-wedge design used in this study. Multilevel modeling allows for the examination of individual-level and cluster-level effects, capturing the hierarchical structure of the data. Regression models will be adjusted for relevant covariates to account for potential confounding factors.

### Interim analyses {21b}

Not applicable. As Table 1 shows, the trial duration is very short. Due to the stepped-wedge methodology and the phased implementation in the different clusters, interim analyses are not planned. As this was considered to be a low-risk intervention, no formal stopping rules are implemented. However, as explained in item 22, the occurrence of serious adverse events is recorded and discussed in the trial steering committee, which would be able to stop the trial if necessary.

### Methods for additional analyses (e.g., subgroup analyses) {20b}

Additional analyses, such as subgroup analyses, will be conducted to explore potential effect modifiers and assess intervention effectiveness across different subpopulations. Subgroup analyses will involve stratifying the sample based on relevant demographic and clinical characteristics. Interaction terms will be included in the regression models to test for effect modification. Subgroup-specific estimates will be reported, allowing for a comprehensive understanding of intervention effects within specific subgroups. Furthermore, predictors for hazardous substance use will be examined using multilevel logistic regression.

### Methods in analysis to handle protocol non-adherence and any statistical methods to handle missing data {20c}

To address protocol non-adherence, an intention-to-treat analysis approach will be employed, where participants are analyzed according to their randomized group assignment, regardless of the intervention received. Additionally, per-protocol analyses will be conducted to assess the robustness of the findings. In handling missing data, multiple imputation [6] will be utilized if necessary. Multiple imputation accounts for the uncertainty associated with missing data by creating multiple plausible imputed datasets. These datasets are analyzed separately, and the results are combined using appropriate rules to provide valid statistical inference. Sensitivity analyses will be conducted to assess the impact of missing data assumptions on the study findings.

### Plans to give access to the full protocol, participant-level data, and statistical code {31c}

This is not applicable because considering sensible topics (trauma, substance use), there are no plans to give full access to the full dataset.

## Oversight and monitoring

### Composition of the coordinating center and trial steering committee {5d}

To conduct and support the trial, different groups are involved in the research project:

1. The *project management group* at the coordinating center is the research team of Esslingen University of Applied Sciences, consisting of the PI, investigators, and research assistants. The coordinating center is responsible for project management and will meet once a week for the duration of the trial to review the trial conduct. Additional meetings will be held in case of the reporting of serious adverse events.
2. The *trial steering committee* has the strategic control about the project and is composed of the researchers in the coordination center, psychologists, social workers, and refugees and also includes public stakeholders from local social welfare and the public health department. The trial steering committee will meet at the start and at the end of the trial. Additionally, meetings will be held on invitation of the project management group in case of serious adverse events and/or challenges to the trial conduct during the course of the trial.
3. The *organizational support groups* are involved in running the trial day-to-day and provide organizational support on each of the nine trial sites. These groups are composed of the staff (social workers and psychologists) in the shared accommodations.
4. The *scientific advisory board* consists of researchers from other universities, politicians and decision-makers from health care sector on national level, experts for substance use prevention on national and federal level, a representative of the social welfare office on a municipal level, and refugees from refugees’ self-organizations. The scientific advisory has met in advance in the planning phase of the trial and discussed the study design and ethical aspects. Additionally, it will be consulted by decision of the trial steering committee in case of challenges in the trial conduct and/or serious adverse events.

### Composition of the data monitoring committee, its role and reporting structure {21a}

This is not applicable because there is no data monitoring committee.

### Adverse event reporting and harms {22}

The overriding principle of the research project is to exclude or minimize the risks for the refugees participating in the surveys. For this reason, there is a close cooperation with the social workers on site of the shared accommodations and their management to provide assistance plans and help services for unforeseen events during the survey (counselling services, educational and psychological support, etc.). In addition, a psychological background service is installed and will be available to deal with psychosocial crises and trauma-related disorders that occur during the data collection. This psychological background service is led by a psychologist and psychotherapist who has a special qualification in dealing with mental health crises.

Possible adverse events among refugees might be the occurrence of clinically relevant problems (e.g., flashbacks in the context of a trauma disorder, or suicidal tendencies). In this case, the local support system will be activated, and, if necessary, the background service will be contacted, or other support systems (e.g., psychiatric emergency services) will be activated.

Furthermore, refugees might experience stress caused by the perceived similarity of the survey process to official appointments as part of their asylum process. That is why providing information about the trial, how to participate, and the fact that it will not have anything to do with asylum decisions is very important. Therefore, information material will be provided in different languages (see above).

It is assumed that the risks for the workforce participating in the study are no higher than corresponding situations that occur in everyday professional life. However, if there are indications of severe stress, advice is given on site; in the event of exceptionally high stress, the background service is consulted.

Any risks that arise are documented by the researchers on site or by the organizational support groups on a standard form and immediately made available to the project management group at the coordinating center and to the trial steering committee. This standard form was developed in advance as part of the ethical clearing process. After this ethical clearing process, the Alice Salomon University Ethics Committee in Berlin (file reference: 01–2024/62) provided ethical approval for this study and considered the intervention as low risk.

In the event of serious adverse events, the project management group decides on the next steps. Indicators of exceptionally high stress (such as panic attacks, suicide attempts, or completed suicides) are reported directly to the members of the steering committee by the project management group. In the event of trial-related serious adverse events, the trial steering committee decides whether to discontinue the study. In that case, the scientific advisory board will also be conducted. The criterion for discontinuing the study is defined as an unreasonable burden on the participants as determined by the trial steering committee. This is decided by a majority vote at an extraordinary meeting on the basis of the available risk documentation. The convening of the extraordinary meeting of the steering committee is convened by the project lead.

### Frequency and plans for auditing trial conduct {23}

The project management group will meet once a week for the duration of the trial to review the trial conduct and perform data monitoring. An independent data monitoring committee will not be established as the intervention was considered to be low risk (see item 22). In case there are protocol amendments organizational support groups, trial participants and the (external) ethics committee that granted ethical approval will be informed.

### Plans for communicating important protocol amendments to relevant parties (e.g.,* trial participants, ethical committees) {25}*

In case of necessary protocol amendment, the project management group will inform the trial steering committee and the shared accommodations. Also, the ethics committee that provided ethical approval of the original protocol will be informed. Any deviations from the protocol will be fully documented. The funder will be informed during regular reports.

### Dissemination plans {31a}

Trial results will be published in an open-access journal and presented at national and international conferences. Further on, there will be short publications about the project and some trial results in easy language and different languages to be accessible for refugees.

## Discussion

This study will be one of the first trials to focus on evaluating participatory prevention concepts for refugees and to generate evidence about effective prevention concepts in shared accommodations. Because of their special social and structural vulnerability, refugees are at higher risk for hazardous substance use. Evidence-based, participatory prevention concepts are needed to address the target-group-specific risk factors and socio-structural challenges to which refugees are exposed to. The participatory multi-component prevention concept provides diverse information and might increase the knowledge of refugees and the multidisciplinary workforce in shared accommodations about hazardous substance use, health literacy, and self-determined use of substances.

Evidence-based prevention concepts are highly important and can be seen as quality standard for effective health care. They might be helpful for reducing structural health inequalities and making the health care system more accessible for refugees by providing low-threshold and setting-oriented prevention measures. Since there are high barriers for refugees to substance use intervention or interventions for refugees are not available at all, the developed and evaluated prevention concept could make the existing care gap slightly smaller and apply at an earlier stage after refugees’ arrival to Germany and during their stay in shared accommodations.

The methodology of the study, the stepped-wedge design, is adequate for the trial setting and will ensure that data on individual as well as accommodational level will be generated. Thus, the focus can also be on structural aspects and on the setting, in which refugees live. Further on, the duration of effects can be estimated using this methodology. So, differences to the accommodations and of how long the concept has been implemented yet can be analyzed. In addition, with the RDS method, the multilingual application, the field experience of the research team, and the participatory nature of the concept, it is assumed that many participants will be reached even though hazardous substance use might be a taboo topic. Nevertheless, this trial will likely have some limitations regarding the sampling. Although we expect to reach the estimated sample, there might be distortions that often entail the RDS method, which is not a traditional sampling strategy, but suitable in non-clinical settings, where access to possible participants might be difficult for researches, but participants’ communities are well networked with one another. Consequently and despite the possible distortions, it is the appropriate method to reach hard-to-reach samples [35] that refugee populations might be. Moreover, international transferability might be limited because refugees are housed differently in different hosting countries, so results from Germany might not be generalizable one-to-one to other countries. Despite this, trial results can be disseminated and adapted to other settings.

## Trial status

Protocol version 2, 08/2024.

Recruitment will begin in 06/2024; data collection will be completed in 10/2024.

## Data Availability

The final trial dataset will only be available for the research team.

## Abbreviations

DASS-21: Depression Anxiety Stress Scale, Version with 21 items
DDPPQ: Drug and Drug Problems Perception Questionnaire
DSM-5: Diagnostic and Statistical Manual of Mental Disorders, Fifth Edition
eHeals: eHealth Literacy Scale
GAF: Global Assessment of Functioning Scale
ICF: International Classification of Functioning, Disability and Health
MATE-Q: Measurements in the Addictions for Triage and Evaluation Questionnaire
OCDUS-5: Obsessive Compulsive Drug Use Scale-5
PraeWi: Name of the research and development project; acronym for the German project title “Präventionsmaßnahmen und Wissenstransfer innerhalb der Sozialen Arbeit bezüglich riskanten Substanzkonsums für Menschen mit Fluchterfahrungen in Übergangswohnheimen“
RDS: Respondent Driven Sampling
SAAS: Substance Abuse Attitude Survey
